# Burdened parents sharing their concerns for their children with the doctor. *The impact of trust in general practice: a qualitative study*

**DOI:** 10.1080/02813432.2019.1639907

**Published:** 2019-07-16

**Authors:** Marit Hafting, Frøydis Gullbrå, Norman Anderssen, Guri Rørtveit, Tone Smith-Sivertsen, Kirsti Malterud

**Affiliations:** a Regional Centre for Child and Youth Mental Health and Child Welfare, NORCE Norwegian Research Center, Bergen, Norway;; b Research Unit for General Practice, NORCE Norwegian Research Center, Bergen, Norway;; c Department of Psychosocial Science, University of Bergen, Bergen, Norway;; d Research Group for General Practice, Department of Global Public Health and Primary Care, University of Bergen, Bergen, Norway;; e Research Unit for General Practice and Section of General Practice, Department of Public Health, University of Copenhagen, Copenhagen, Denmark

**Keywords:** General practitioner, children of impaired parents, trust, qualitative analysis, focus groups

## Abstract

**Objective:** The aim of this study was to recognise the preconditions experienced by general practitioners (GPs) in addressing the children’s needs when ill and substance abusing parents consult for their own health problems.

**Design:** Qualitative analysis of 38 case stories told by GPs in focus group interviews.

**Setting:** Focus group interviews of four continuing medical education groups for GPs in western Norway.

**Subjects:** 27 GPs (nine females) with at least 5 years’ experiences in general practice.

**Results:** Different aspects of the GPs’ perceived mandate of trust from the parents was a precondition for the children’s situation to be addressed. In some case stories the participants took an open mandate from the parent for granted, while in others they assumed that the parent did not want to discuss their family situation. Sometimes the participants had faith that by continuing with their ordinary GP tasks, they might obtain a more open mandate of trust. Their evaluation of the mandate of trust seemed to impact on how the GP could adopt a mediating role between the parents and various support agencies, thus supporting children who were at risk.

**Discussion/conclusion:** The children most at risk may remain invisible in GPs’ encounters with their parents, possibly because their parent’s health problems and overall situation overshadow the children’s situation. The mandate of trust from burdened parents to GPs can be a fruitful concept in understanding the interaction regarding the welfare of the parent’s children. Negotiating the mandate of trust with parents by explicitly addressing trust and having an ongoing discussion about the mandate and its limits might be an option to secure the children support if necessary.

**KEY POINTS**
Offering children of burdened parents information and support can be crucial for health promotion and illness prevention.

- A general practitioner’s (GP’s) evaluation of the trust parents have in them can determine the extent of support children receive.
- Depending on the parents’ level of trust, GPs may take a mediating role between support services and parents for the benefit of the children.
- A negotiation concerning the trust parents have in the GP may open up possibilities for GPs to offer children necessary support.

## Introduction

Children of parents with serious health and psychosocial problems are often vulnerable. They struggle with daily challenges more often than other children do, and they are at risk of developing health and social problems [1–3]. For these children, health promotion and illness prevention are important.

In Norway, a new legislation was launched in 2010 [4]. According to this law, all healthcare providers who encounter parents with mental health problems, severe physical health problems or substance abuse problems are obliged to ensure, with the parents’ informed consent, that children receive information about their parents’ health problems and are given support, if necessary. Families with these health problems face different challenges related to medical, practical and cultural issues. However, children within these families also face similar challenges due to the risk of reduced parental capacity to meet the needs of the children [5,6]. Studies describing the special needs of children as next of kin are multiple [7–9], but studies about the potential contributions of general practitioners (GPs) are few [10,11].

When burdened parents consult GPs in relation to their own problems, the consultation might prove a starting point for a discussion about their children’s situation [12]. However, providers face barriers in addressing the children’s problems [13–15]. Previous sub-studies [14,16,17] have indicated that GPs are in a good position to identify at-risk children and ensure them follow up in relation to their needs, but they have often missed opportunities to do so because of structural and relational barriers. These barriers comprised limitations present in the framework of general practice: short consultations, time pressures and different family members enlisting with different GPs. Many GPs also feared jeopardising the doctor–patient relationship, offending the patient or placing even more burdens on them.

Furthermore, these sub-studies demonstrated that their children’s situation might be a sensitive topic for parents. When parents consult GPs regarding their own health problems, it may be difficult for the GP to know whether they will permit a discussion about their children’s situation or feel offended by the GP introducing the topic. According to Fugelli [18] and Skirbekk [19], a patient’s trust in a professional is often a precondition for an engagement in conversations concerning sensitive issues. In the present study, we address a specific aspect of the relationship between GPs and burdened parents: the impact of trust.

## Mandate of trust

Skirbekk et al. conceptualise *trust* as the patient’s willingness to accept the doctor’s judgement in matters of concern to the patient, usually as an implicit agency in the encounters [20]. The patient may allow for a broader or more limited scope concerning the topics discussed in the consultation. Skirbekk et al. introduce the concept *mandate of trust* as “the degree of openness, and in what areas the physician is authorized to exercise his or her judgement in matters of concern to the patient” (p. 1184). The authors found that consultations could be adequately performed based on a *limited mandate* of trust from the patient, when the patient’s concern was quite specific and when the patient and the doctor had a similar understanding of the problem presented. Patients with more composite complaints and chronic illnesses as well as multifactorial and ambiguous symptoms often emphasised the need for an *open mandate* of trust with the GP as a premise for discussing their concerns. Skirbekk et al. found that the provision of an open or limited mandate of trust by the patient depended on the doctor–patient relationship and the complexity of the problem. Based on this, we assume that the GP will usually need an open mandate of trust from the patient to introduce sensitive topics, such as concerns about the patient’s children, in the consultation. The concept of the mandate of trust will support our analysis of how GPs identify and ensure support for their patients’ children during consultations [21].

## Objective

The aim of this study was to recognize preconditions experienced by GPs in addressing the children’s needs when ill and substance abusing parents consult for their own health problems.

## Materials and methods

Data stem from focus group interviews taken from a broader research project concerning GPs’ reflections and experiences in dealing with the special needs of children as next of kin. Details concerning the design, methods and materials of the original project are reported elsewhere [14] and will be briefly summarised here. Specific methodological information regarding the aim and process of the secondary analysis reported in this article will be included.

### Focus groups: sample and interviews

For this project, it was necessary to include GPs who had some experience with the topic at hand; thus, we established a purposive sample [22] of 27 GPs (nine females) with at least 5 years’ experience. Four focus group interviews with five to nine participants were conducted. When GPs meet to discuss a clinical subject, they tend to tell case stories [23]. The GPs participating in the focus groups were encouraged to bring relevant stories from their practice to the table. These stories form the units of the secondary analysis conducted for the present article.

The focus group interviews were moderated by the second author (FG) and supported by the first author (MH). We invited the participants to share cases stories from their practices and reflections on these and the other participants’ case stories. We asked for their experiences when speaking to ill parents about their children’s situation, talking with children about their parent’s problems and collaborating with service workers and support services concerning at-risk children. The participants contributed to the discussion with case stories from their own practice, including case stories they had prepared beforehand and case stories remembered during the discussion. The concept of mandate of trust was not raised as a topic in the discussions, by neither the GPs nor the moderators. Out of the 27 focus group participants, 19 GPs (eight females) contributed case stories; most of these 19 participants contributed two case stories, with others contributing between one and four case stories. For more details about the sample, data collection, primary analysis and previous findings, see Gullbra et al. [14].

### Analysis

The analysis was guided by the research question and supported by Skirbekk et al.’s “mandate of trust” concept [20]; we aimed to analyse cases where trust and the mandate of trust was an issue. We read the transcript of the interviews as a whole and identified 38 case stories concerning burdened parents and their children, from which we performed a thematic analysis [24] of the text material. First, two authors (MH and FG) read all case descriptions together, identified situations where trust was a topic and gave these situations descriptive codes. Then MH compared these codes and established subordinate and overarching themes that were interpreted in the context of the entire data set from the focus groups. In addition, in a third order interpretation the theoretical concept of mandate of trust was explicitly imposed upon data, our findings situated visibly in the Results section. The thematic analysis and the third order interpretations were led by MH. All authors (KM, FG, NA, TSS and GS) participated in several in-depth discussions of ideas and drafts.

### Research ethics

The Regional Committee for Medical and Health Research Ethics, Western Norway, stated that the Act does not apply to this project. Participants signed an informed consent form.

## Results

The analysis demonstrated different aspects of the GPs’ perceived mandates of trust from the parents as preconditions for addressing the children’s situation. In some of the case stories the participants took an open mandate of trust from the parent for granted and therefore engaged in the children’s situation. Or, they had faith that by continuing their ordinary GP tasks in the family, they might obtain a more open mandate of trust. In some cases, the GPs seemed to expect that the parent did not want to discuss the family situation and therefore did not raise the issue. Here the GPs either had concerns for the children and followed them by ad hoc consultations or collaboration with relevant agencies, or they solely concentrated their efforts to secure continuity of care for the burdened parent. Furthermore, how they perceived the mandate of trust seemed to have an impact on how the GPs could adopt a mediating role between the parents and various support services. In situations indicating that participants might possess an open mandate from the parents, they could assist at-risk children. No participants reported explicitly discussing the doctor–patient relationship with the patient in order to evaluate how the patient would feel if their children’s situation were discussed. Below, we present our main findings that are based on how we interpreted the impact of the mandate of trust.

### The GP could talk about the children’s situation due to a perceived open mandate of trust from the parents

In some case descriptions, the GP described how they directly addressed the children’s situation during the consultation, and the parents allowed it. In some cases, they said that the parents spontaneously brought up the topic and asked the GP for advice and involvement. Our interpretation is that the GP in these cases took an open mandate of trust for granted. In other cases, however, the GPs stated that they were aware of the children’s possible needs but handled different issues on request from the burdened parent before they gradually addressed the children’s well-being. An example of this was a female GP who spoke about a young mother with terminal cancer. She had two boys and was in conflict with her ex-husband. The GP gradually got to know the patient’s parents, children, ex-husband and various helpers:

‘When she got ill, her reaction was denial over a long period. Gradually, we had good conversations. I could give her advice about how to talk to the children, etc. I was there for home visits sometimes and met a health professional (…) who could be there more than I could. (…) The ex-husband contacted me, and I had a good report with him. (…) Gradually, he came more into the family again and, between the parents, it was decided that after her death, the boys should live with him. I also contacted the Cancer Society.*’*

We interpret this to be a case story about a GP who was sensitive to the patient’s capacity to acknowledge the seriousness of the situation. Over time, she laid the foundations for a mandate of trust from the mother to address the family situation.

### The GP deduced that the mandate was too limited to talk about the children’s situation, and kept the children in mind while balancing the relationship with the parents

In other cases, the GPs did not explicitly address their concerns for the children’s well-being or future psychosocial situation with the parents. They had the children in mind, but they did not dare jeopardise the relationship by discussing the children’s situation, since they evaluated this to be a sensitive subject. Some of the GPs said that they did not want to load more difficulties onto the already heavily burdened parents.

A male GP spoke about a family with three children who were, as far as he knew, doing well, and a father whom he also evaluated as doing well. The mother suffered from paranoid psychosis and had had several referrals to psychiatric hospitals. The interviewer asked the GP if he had addressed the children’s situation with the parents. The answer was:

‘In this family, we do balancing exercises. I would be afraid that if the mother had been confronted with a question like that, she would leave my list. Probably that would have created much conflict.’

Here, the GP utilises the phrase “balancing exercises” to describe what we interpret as dealing with a mandate of trust that he found too limited to address the children’s need for information about the mother’s condition.

Some of the cases gave us the impression that it was difficult to achieve a relationship with parents that allowed them to address concerns over the children when the parents appeared to be in denial or had constrained or distorted awareness of their own problems and parental abilities. In addition, it seemed to be more difficult to address the children’s situation when the parents were in conflict with support services.

An illustration of this point is a case a male GP described where the mother suffered from postpartum psychosis following the births of all of her four children. In his opinion, both parents had sparse intellectual resources. During their work with the family, the local child welfare services (CWS) sent the GP questionnaires to fill in. However, there were many questions that he was unable to answer:

‘I see the children only when they have runny noses. Then the parents tell [me] how well functioning the children are. I have been there on [a] home visit once and saw that the children were jumping around like rabbits long after bedtime. The parents said this was a special situation. I cannot take any action, as far as I can see.*’*

In this case, we infer that the GP considered himself to be in a position where he could not build a mandate of trust from the parents to allow him to address his concerns for the children, because of the parents’ denial and idealisation.

In the focus group discussion related to the case presentation, some participants said they had faith that continuing their ordinary GP tasks for the family would eventually allow them to explicitly engage with the children. Meanwhile, they followed the children’s development indirectly from school meetings and consultations for more trivial problems. As the aforementioned GP claimed:

*‘*When these people let you in behind the hood, they share their concerns for the children. They are good people and they want the best, and when they believe that I also do so, I am allowed to participate in talks about these topics also.’

### The GP abandoned hope of obtaining a mandate of trust and did not engage in the children’s situation

These cases often revealed long-lasting relationships with the burdened parent, and in the discussion, the GPs reflected that they valued the continuity of care as crucial for the vulnerable patient. In these cases, it appears that the GPs indirectly chose to prioritise the health and welfare of the parent over the children. One male GP spoke about a father with substance abuse problems who had four children. He had frequent consultations with the patient:

‘I concentrate my efforts to give as good medical care as possible to the father. I cannot imagine how to talk to him about the children. I am worried, but I consider this worrying as part of being a GP. You are an observer to adversities, and often you cannot interfere.’

Here, our interpretation is that the GP does not try to establish the foundations for the mandate of trust needed to explore the children’s situation. He justifies this with a matter-of-fact attitude: As a GP, you cannot solve all of the problems you come across.

### An open mandate of trust may facilitate a mediating role for the GP between parents and multidisciplinary collaborators

Several of the case descriptions contained details about the patients’ local communities. The participants had comprehensive knowledge of the local society, with many of them living in the community themselves. They took part in football matches for children and school arrangements and could observe how the children of their patients were coping. In these accounts, the participants also revealed knowledge about actual professional collaborators. Some participants collaborated in preventive care for children with health visitors. Other case descriptions, including both rural and urban communities, described how the GPs had only a “peephole” into their patients’ everyday life and social situation, but that this “hole” gradually widened through their long-lasting relationship with the patients. Subsequently, they gained insight into the other services and personnel involved in supporting the families, such as school psychology services and CWS.

A female GP described a case of a mother with small children. The mother had paranoid thoughts and believed that she might hurt her children:

‘I had some talks with her and referred her to the adult psychiatry outpatient clinic. They had some collaboration with child psychiatry. After a while, they contacted the local health visitor. She has followed up the family together with me, and the whole situation has calmed down. This is a good example of how many agencies can work together.*’*

Our interpretation here is that the GP described a situation where she took for granted an open mandate from the mother to collaborate with various agencies to find suitable solutions for the family.

However, several case descriptions revealed problems in relation to cooperation between the family, GPs and primary health care, schools, hospital-based services and the CWS. The GPs sometimes became the parents’ allies. They were the only professional who interacted with the family over a long period of time. From this position, they could gradually find collaborators that the family and they themselves had confidence in, thus building a professional network around the children. A male GP recalled a case about a family he had dealt with for 20 years. The mother suffered from anxiety and was often in conflict with authorities:

‘My experiences with specialist health ward concerning this mother was not good, and the school and pedagogic support services had handled this family in a wrong way. The mother thought that they were persecuted by the support services. (…) The youngest daughter refused to go to school, because the mother said it was dangerous there. Then we had to mobilize the CWS. I still take part in a structured multidisciplinary network. I think they [the family] experience it as reassuring that I am there, because I have listened to them and supported them all the time.*’*

An interpretation of this case story is that the GP perceived an open mandate of trust from the parents. He succeeded in achieving a limited mandate from the parents to other important agencies supporting the children. The parents did not have enough trust in the other agencies to give them an open mandate. Thus, the GP was needed because the parents relied on his evaluation of what was best for them.

## Discussion

We utilised the “mandate of trust” as a perspective from which to analyse case descriptions about how GPs address children’s needs in encounters with burdened parents. This strategy gave access to reflection upon aspects in the consultation process. The mandate of trust perceived by the GP could determine the GP’s approach in supporting the parents and children in consultations and collaborations. This means that the same GP might use different approaches towards families depending on how s/he perceived the mandate of trust they gave. Achieving the necessary trust from parents could be difficult, especially in cases that were more complex. In these cases, most GPs considered their opportunities to support the children as being limited, and their support for the family was confined to securing medical services for the parent.

### Strengths and weaknesses

Although the focus group participants were recruited with an eye towards variety (women and men, urban and rural, solo practices and group practices), other important influencing factors were not represented, including the GPs’ additional work experiences, family status or political affiliation. For instance, GPs taking part in preventive health services for children tend to be more aware of children’s situations and have a deeper knowledge of local support networks for children [25]. We assume that the same can be said for GPs with their own young children.

The participants were recruited from established continuing medical education groups, and they knew each other well. Because of peer pressure, they might have wanted to speak of success stories and speak less about cases where they did not contribute or failed [23]. This scenario might have been strengthened by the fact that first interviewer was a GP (FG), the other a child and adolescent psychiatrist (MH) and that the overall aim of the research project was to obtain knowledge on how to support the children of burdened parents [26]. However, we deliberately asked for a variety of experiences and made an effort to establish an atmosphere that was not judgemental. In addition, a third of the case descriptions concerned cases where the participants had only a limited engagement in the children’s situation.

The primary analysis of the focus group interviews focused on the GPs’ thoughts and experiences [14]. We conducted a secondary analysis to emphasise the potential implications of a mandate of trust, searching for case descriptions where trust might be an issue. This selection process ran the risk of confirmation bias, where predetermined notions become strengthened and other possible interpretations become neglected. However, we tried to overcome such bias by deliberately searching for alternative explanations in the text, such as assuming an impact was as a result of the frames of general practice or the impact of the personal situations of GPs.

Our data comprised descriptions of how the GPs remembered and chose to present the actual case stories. We had no observations from interactions or events, and do not know how the corresponding patients evaluated the encounters. More specifically, we have no direct information about what mandate of trust they wanted to give the GP. There might have been a mismatch between how the GPs evaluated the situation and what the parents actually wanted. From a previous study [16], we learned that parents usually want the GP to address the children, but they have to be prompted in this. Furthermore, the mandate of trust was a theoretical concept introduced by us during analysis; it was not mentioned explicitly by the participants. Because of this, we carefully tried to attend to intersubjectivity by highlighting the interpretative positions from which our findings were developed.

### Explicit negotiations concerning trust - an option?

Trust is an important quality of every patient–doctor relationship [18,27]; it is usually taken for granted or implicit in the relationship [20,28]. In the case descriptions, the focus group participants did not report that they explicitly addressed trust as a topic in their encounters with the patients.

There are many reasons why it is important for GPs to raise parenting issues in encounters with burdened patients. Such a dialogue could be conceptualised as a negotiation. Studies have revealed that parents struggling with various health problems are worried about their children and want professionals to bring up the topic [16,29]. At the same time, parents typically want to appear responsible and able to cope in challenging situations [16]. The GP does not know the parent’s attitude without asking, and if the GP explicitly addresses the situation of the children, the relationship may be jeopardised [30]. Posing such an explicit and sensitive question might lead to open distrust, a limited mandate of trust or an open mandate to discuss the children.

Sometimes the parent is worried about losing custody of their children [17,31]. This is a relevant concern, because Norwegian legislation [32] orders that GPs report to CWS if they have serious concerns about a child’s living conditions. CWS, however, provides various types of resources to vulnerable families, and the GP may, in relevant cases, use the opportunity to lay the foundations for a productive relationship between the parents and CWS.

A negotiation process regarding the mandate of trust could be initiated by the GP by asking the parent for general permission to address vulnerable topics; the parent is then given the chance to regulate the interaction. In being asked for permission, the parent may perceive respect and trust from the GP, which may increase the likelihood of a process towards a more open mandate of trust. Many parents will accept the GP raising sensitive issues, and the GP may then carefully ask about possible challenges related to the children’s well-being. In cases where a parent says no to the GP, the GP will have to accept this in the consultation, but s/he must keep the children in mind and perhaps try to bring them into the discussion at a later encounter. In addition, s/he can follow their development in collaboration with other instances like health nurses and schools.

A well-functioning collaboration between parents and professionals such as teachers, health nurses, psychologists, CWS, GPs and other helpers is of significant importance to the quality of life and future health of the children [33]. The GP can negotiate with the parents if their mandate also includes that the GP adopts a mediating role between them and different support services.

### Is it possible for the GP to reach the most invisible and vulnerable children?

Skirbekk [20] argue that a mandate of trust from the patient depends on the complexity of the case and the doctor–patient relationship. Our case descriptions supported this and demonstrated challenges with the GP obtaining a mandate of trust from burdened parents with comprehensive co-morbidity of somatic, psychiatric or substance abuse and denial of the problems. If parents constantly change their GPs and move frequently, it leads to a risky situation for the children as next of kin, as they might not be identified as being at-risk [14,17]. In such living situations, the GPs often were anxious about losing the rapport with the parents. These children are at risk of becoming “invisible” children [11,14,34,35], and some of them live under adverse conditions. Such limitations in the GP’s working conditions must be conveyed to collaborators and policymakers. Less successful experiences may be suppressed or under-communicated in a discourse where the prevailing matter is the GP’s influential position and responsibility. GPs should always keep these children in mind; they can gain perspective on the children’s situation in various ways, including ad hoc consultations with the children and through multidisciplinary forums. They can also search for other professionals who may be supportive [36].

## Conclusion

At-risk children may remain invisible in encounters between their parents and GPs, possibly because their parent’s health problems and situations overshadow the children’s situation in a busy GP day with structural and relational limits. The mandate of trust from a burdened parent to a GP can be a fruitful concept in understanding the interaction regarding the welfare of the parent’s children. Our analysis has demonstrated how several factors influence the extent of such a mandate. Negotiating the mandate of trust with the patient by addressing trust explicitly and having an ongoing discussion about the mandate and its limits might be an option to make the children visible in the encounters.
